# Sex-Dependent Effects of Exogenous Irisin on Pancreatic Endocrine-Cell Immunophenotype in Post-Weaning Wistar Rats

**DOI:** 10.3390/cimb48090967

**Published:** 2026-09-21

**Authors:** Cezary Osiak-Wicha, Małgorzata Manastyrska-Stolarczyk, Siemowit Muszyński, Katarzyna Kras, Aleksandra Dajnowska, Ligia Janicka, Katarzyna Woźniak, Ewa Tomaszewska, Tymoteusz Słowik, Piotr Dobrowolski, Marcin B. Arciszewski

**Affiliations:** 1 Department of Animal Anatomy and Histology, Faculty of Veterinary Medicine, University of Life Sciences in Lublin, Akademicka 12, 20-950 Lublin, Poland; cezary.wicha@up.edu.pl (C.O.-W.);; 2 Department of Biophysics, Faculty of Environmental Biology, University of Life Sciences in Lublin, Akademicka 13, 20-950 Lublin, Poland; 3 Department of Animal Physiology, Faculty of Veterinary Medicine, University of Life Sciences in Lublin, Akademicka 12, 20-950 Lublin, Poland; 4 Experimental Medicine Center, Medical University, Jaczewskiego 8, 20-090 Lublin, Poland; 5 Department of Functional Anatomy and Cytobiology, Maria Curie-Sklodowska University, Akademicka 19, 20-033 Lublin, Poland

**Keywords:** insulin, glucagon, somatostatin, proliferation, apoptosis

## Abstract

Background/Objectives: Irisin is an exercise-associated myokine with reported effects on β-cell survival, insulin secretion, and pancreatic tissue protection in experimental models of metabolic stress. However, less is known about its influence on the developing endocrine pancreas under physiological conditions. Accordingly, the aim of the present study was to determine whether exogenous recombinant FNDC5/irisin is associated with changes in pancreatic endocrine-cell immunophenotype and islet-level markers of cell turnover in male and female Wistar rats. Methods: Thirty-two 3-week-old Wistar rats were assigned to control and irisin-treated groups, with males and females analyzed searately. Irisin was administered intraperitoneally at 100 µg/kg body weight once weekly for four weeks. Control animals received 0.9% NaCl. At the end of the experiment, serum cholesterol, fructosamine, and lipase were measured as supportive biochemical endpoints. Pancreatic sections were analyzed immunohistochemically for insulin, glucagon, somatostatin, PCNA, and cleaved caspase-3. Results: Irisin treatment was not associated with statistically significant changes in serum cholesterol, fructosamine, or lipase. In males, irisin increased insulin immunoreactive area, insulin-positive cell percentage, and insulin-positive cell density. In females, irisin decreased glucagon immunoreactive area and glucagon-positive cell density, whereas glucagon-positive cell percentage remained unchanged. Somatostatin immunoreactive area decreased after irisin administration in both sexes, while somatostatin-positive cell percentage and density increased in males and females. PCNA-positive cell percentage decreased in both sexes after irisin treatment. Islet cleaved caspase-3 immunoreactivity, assessed as optical density, was not significantly altered. Conclusions: Exogenous irisin modified pancreatic endocrine-cell immunophenotype in a sex- and cell-type-dependent manner in post-weaning rats. These changes occurred without significant alterations in serum lipase, fructosamine, or cholesterol and without a detectable increase in islet cleaved caspase-3 immunoreactivity under the applied analytical conditions.

## 1. Introduction

Skeletal muscle acts as an endocrine organ that communicates with distant metabolic tissues through myokines [1,2]. Irisin is a peptide hormone generated by proteolytic cleavage of fibronectin type III domain-containing protein 5 (FNDC5), a transmembrane protein whose synthesis is strongly linked to peroxisome-proliferator-activated receptor gamma coactivator 1-alpha (PGC-1α) activity in skeletal muscle. It was initially characterized as an exercise-associated myokine that promotes browning of white adipose tissue, increases thermogenic gene expression, and improves systemic glucose homeostasis. Although skeletal muscle is regarded as the principal source of circulating irisin, FNDC5/irisin expression has also been demonstrated in adipose tissue and several other organs, indicating that irisin may participate in broader endocrine and paracrine signaling networks. Mechanistically, the best characterized irisin receptor system identified to date involves αV-class integrins, which provides a plausible framework for its pleiotropic actions in metabolically active tissues [3,4,5,6].

Beyond its physiological association with exercise, exogenous irisin has attracted attention as a biologically active intervention in experimental models of metabolic dysfunction. In pancreatic β-cells, recombinant irisin has been reported to enhance insulin biosynthesis and glucose-stimulated insulin secretion, stimulate cell proliferation, and attenuate apoptosis induced by glucolipotoxic, lipotoxic, or oxidative stress conditions. These effects have been linked to signaling pathways involving AKT, ERK, p38 MAPK, AMPK, and redox-sensitive mediators, and are accompanied by reduced expression of pro-inflammatory or pro-death molecules in stressed β-cells. Protective actions of irisin have also been described in pancreatic injury models, including chronic and acute pancreatitis, where it reduced tissue injury, inflammation, oxidative stress, fibrosis, or neutrophil extracellular trap formation. Taken together, these findings support the view that exogenous irisin can influence pancreatic homeostasis, but most available evidence comes from disease-oriented models or β-cell-focused systems rather than from integrated histological assessment of the whole pancreas in vivo [7,8,9,10,11,12].

The pancreas is a particularly relevant target in this context because available data indicate that irisin is not only systemically delivered to the gland but may also participate in local islet biology. FNDC5/irisin has been detected in rat and human pancreatic islets, including both α- and β-cells, and isolated human islets have been shown to secrete irisin in a glucose-dependent manner. At the same time, systemic irisin administration in rats was found to modify islet blood flow, suggesting that pancreatic responses to irisin may involve both endocrine-cell effects and changes in the local microenvironment. This makes the pancreas a biologically plausible site in which exogenous irisin could alter endocrine-cell organization, secretory balance, and tissue homeostasis, even in the absence of overt diabetes or pancreatitis [13].

In the present study, this rationale was examined by immunohistochemical assessment of pancreatic markers representing the principal endocrine-cell populations and selected indices of cell turnover. Immunostaining for insulin, glucagon, and somatostatin allowed evaluation of β-, α-, and δ-cell compartments, which together define the core endocrine architecture of the islet and its local paracrine regulation. This approach is relevant because glucagon and somatostatin are integral components of intra-islet signaling, and changes in their distribution may accompany altered β-cell status rather than changes in insulin immunoreactivity alone. Proliferating-cell nuclear antigen (PCNA) and cleaved caspase-3 were included to assess proliferative activity and apoptosis, respectively, in line with previous evidence that irisin may affect β-cell survival and replication. This framework is further supported by evidence that endocrine pancreas biology differs between males and females, justifying analysis in both sexes. Accordingly, the aim of the present study was to determine whether exogenous irisin is associated with changes in pancreatic endocrine-cell immunophenotype and selected indices of proliferation and apoptosis in male and female Wistar rats [14].

Given that pancreatic endocrine maturation continues during the postnatal and post-weaning period, the use of young, post-weaned rats provides a useful model for assessing whether exogenous irisin can influence the pancreas during a developmental window in which islet structure and functional competence are still being refined. In rodents, weaning is a critical transition for β-cell maturation, with major changes in glucose responsiveness, gene-expression programs, and stimulus–secretion coupling occurring during this stage rather than being fully established at birth. On this basis, we hypothesized that exogenous recombinant FNDC5/irisin administration in post-weaned Wistar rats would modify pancreatic islet phenotype, as reflected by changes in insulin, glucagon, and somatostatin immunoreactivity, and would affect islet-level PCNA and cleaved caspase-3 immunoreactivity.

## 2. Materials and Methods

### 2.1. Animals and Experimental Design

The experiment was conducted at the Experimental Medicine Center of the Medical University of Lublin after approval by the Local Ethics Committee for Animal Experiments (approval no. 31/2024). All procedures were performed in accordance with applicable national and international regulations for the care and use of laboratory animals and in line with Good Laboratory Practice principles.

The study included 32 post-weaning Wistar rats (*Rattus norvegicus*), 3 weeks of age at the beginning of the experiment. This developmental stage was selected because it represents an early postnatal period of intensive growth and tissue maturation, allowing assessment of the effects of exogenous irisin during a still immature stage of pancreatic development. The animals were obtained from the Experimental Medicine Center of the Medical University of Lublin (Lublin, Poland). Animals were housed four per cage in same-sex cages. Each cage contained animals from only one treatment group. Within each sex/treatment group, animals were distributed across two cages. Before the start of the experiment, animals were allocated to cages by staff members of the Experimental Medicine Center, and cages were then randomly assigned to the control or irisin-treated group. Personnel involved in treatment administration were not involved in immunohistochemical quantification or statistical analysis. Before microscopic evaluation, tissue sections and digital images were coded, and the observer performing quantitative image analysis was blinded to both sex and treatment group. The allocation code was decoded only after completion of image analysis and statistical processing. The animals were housed under standard environmental conditions, with controlled temperature and humidity, a 12 h light/12 h dark cycle, and ad libitum access to standard laboratory chow and drinking water. Animal welfare was monitored throughout the experiment. All procedures were performed by trained personnel in accordance with the approved experimental protocol. Intraperitoneal injections were performed using aseptic technique and appropriate restraint to minimize stress and procedure-related discomfort. Animals were monitored daily for general condition, behavior, posture, grooming, food and water intake, locomotor activity, and signs of pain or distress. The number of animals per experimental group (n = 8 per sex/treatment group) was established with reference to previously published in vivo studies using comparable recombinant irisin administration protocols and was additionally evaluated using Mead’s resource equation for ANOVA-based animal experiments. The final group size exceeded the minimum sample size indicated by this approach while remaining consistent with the reduction principle of the 3Rs [15]. The experiment started in the third week of life and continued until the end of the seventh week, corresponding to a 4-week treatment period.

Rats in the experimental group received recombinant rat FNDC5/irisin protein (Cusabio Technology LLC, Wuhan, China; cat. no. CSB-EP810392RA1), corresponding to amino acids 29–140 of rat FNDC5. According to the supplier’s documentation, the product was expressed in *Escherichia coli*. The sequence corresponds to the complete reported mature/secreted irisin region of rat FNDC5. The protein was dissolved in sterile 0.9% NaCl and administered by intraperitoneal injection at a dose of 100 µg/kg body weight once weekly for four consecutive weeks, always on the same day of the week, for a total of four doses. The dose and administration schedule were selected a priori on the basis of previously published in vivo rodent protocols in which repeated systemic administration of recombinant irisin/FNDC5 at a low cumulative weekly dose of 100 µg/kg produced measurable tissue-level effects. In the present study, this regimen was used as a repeated intermittent exposure protocol during the post-weaning period, rather than as a schedule intended to maintain sustained circulating or pancreatic peptide concentrations. The once-weekly schedule was also selected to limit repeated injection-related handling and stress in young animals [16,17,18,19]. Animals were euthanized one week after the final irisin/vehicle injection by exsanguination from the abdominal aorta under isoflurane anesthesia, and whole pancreases were collected for further analyses. Physical activity was not experimentally manipulated in this study. Animals were housed in standard cages without running wheels or forced-exercise protocols, and spontaneous physical activity was not quantified.

### 2.2. Blood Sampling and Biochemical Analyses

During exsanguination, blood samples were collected for biochemical analyses. Blood collection was performed after a 12 h fasting period, during which the animals had free access to drinking water. After clot formation, the blood samples were centrifuged to obtain serum, which was subsequently used for the determination of selected biochemical parameters. Serum cholesterol, fructosamine, and lipase concentrations were assessed as supportive biochemical endpoints. The analyses were performed as an external laboratory service by Vet Diagnostyka (Lublin, Poland), according to the laboratory’s standard diagnostic procedures.

### 2.3. Sample Collection and Tissue Processing

After euthanasia, pancreatic tissue samples were collected and immediately fixed in 10% neutral buffered formalin for 24 h at room temperature. For each animal, one pancreatic fragment containing endocrine islets was selected from the whole pancreas and processed for paraffin embedding. One paraffin block per animal was used for immunohistochemical analysis. The tissue block was selected on the basis of preserved pancreatic parenchyma, absence of major processing artifacts, and the presence of identifiable islet profiles. Block selection was not based on staining intensity or treatment group. Following fixation, the samples were rinsed in running tap water to remove residual fixative. The tissues were then dehydrated through a graded ethanol series, cleared in xylene, and infiltrated with Paraplast. Subsequently, the samples were embedded in paraffin blocks using a modular tissue embedding system (MYR EC-350, Casa Álvarez Material Científico SA, Madrid, Spain). Paraffin-embedded pancreatic samples were sectioned on a rotary microtome (HM 360, Microm, Walldorf, Germany) into 4 µm-thick sections. The sections were mounted on microscope slides (Thermo Scientific, Menzel-Glaser, Braunschweig, Germany) and used for subsequent immunohistochemical staining.

### 2.4. Immunohistochemical Analysis (IHC)

Immunohistochemical staining was performed on paraffin-embedded pancreatic sections using the DAB chromogen method. Sections were deparaffinized in xylene and rehydrated through a descending ethanol series to distilled water. After washing in PBS, heat-induced epitope retrieval was performed in citrate buffer (pH 6.0) using a pressure cooker (8 min, 120 °C, multicooker RMC-PM381-E, Redmond Industrial Group, Guangzhou, China). The slides were then allowed to cool, and endogenous peroxidase activity was blocked by incubation with 3% hydrogen peroxide for 15 min. After washing in PBS with Tween-20, the sections were incubated with PBS containing Triton X-100 for 10 min and subsequently washed again in PBS with Tween-20. The sections were then incubated overnight at 4 °C with primary antibodies against insulin, glucagon, somatostatin, cleaved caspase-3, and PCNA (Table 1).

On the following day, the sections were washed in PBS with Tween-20 and incubated with a two-step detection system (ImmunoLogic WellMed BV, Duiven, The Netherlands). First, a post-antibody blocking reagent was applied for 15 min, followed by washing in PBS with Tween-20. The sections were then incubated for 30 min with a horseradish peroxidase (HRP)-conjugated secondary detection reagent. After subsequent washing, the immunoreaction was visualized using a 3,3′-diaminobenzidine (DAB) substrate kit (DAB substrate kit; ab64238; Abcam, Cambridge, UK) for up to 2 min. The sections were counterstained with hematoxylin (Patho, Mar-Four, Konstantynów Łódzki, Poland), rinsed in running tap water, dehydrated through an ascending ethanol series, cleared in xylene, and mounted for microscopic evaluation.

For each immunohistochemical reaction, negative-control sections were processed in parallel by omitting the primary antibody and replacing it with PBS. No specific DAB staining was detected in the negative-control sections. The primary antibodies were selected based on their documented reactivity with rat tissue and suitability for immunohistochemical analysis of paraffin-embedded sections. Their use was further supported by previous studies in which the same catalogue-number antibodies were successfully applied in immunohistochemical analyses [20,21,22].

### 2.5. Quantitative Image Analysis

Microscopic evaluation was performed on pancreatic sections after immunohistochemical reactions using a light microscope (BX-51 DSU, Olympus, Tokyo, Japan) equipped with a digital camera (DP-70, Olympus, Tokyo, Japan). Representative high-resolution images of pancreatic islets were captured at 200× and 400× magnifications using Cell^M 2.3 software (cellSens Standard, Olympus, Tokyo, Japan) under constant illumination, exposure, brightness, and contrast settings throughout the analysis. All images were acquired by a single operator under identical imaging conditions to minimize technical variability between samples. Digital images were saved in an uncompressed format and further analyzed using ImageJ software (version 1.54f, National Institutes of Health, Bethesda, MD, USA; https://imagej.net/ij/index.html, accessed 1 June 2026). For each immunohistochemical staining, pancreatic islets were identified morphologically and manually delineated as regions of interest (ROIs) for subsequent quantitative analysis.

For each animal and each immunohistochemical reaction, 10 pancreatic islet profiles were selected by systematic random sampling from three non-consecutive sections separated by 200 µm. Approximately equal numbers of islets were analyzed per section, with three or four islets selected from each section to obtain a total of 10 islets per animal. The initial field was selected randomly, after which the entire section was examined according to a predefined systematic pattern. Consecutive eligible islets were included until the required number was reached. Islets were included irrespective of size when the complete islet profile was contained within the tissue section and image field, the tissue architecture was preserved, and the islet boundary could be clearly identified. Profiles affected by folds, tears, section-edge artifacts, staining precipitates, or excessive background staining were excluded. The same sampling and inclusion criteria were applied to all animals. The total area of each analyzed two-dimensional islet profile was recorded during ROI analysis. Because a single histological section does not determine the true three-dimensional size of an islet, this parameter was referred to as sampled islet profile area rather than total islet size. For each animal, the areas of the 10 analyzed islet profiles were averaged to obtain one animal-level value for mean sampled islet profile area, which was used as a sampling-control parameter.

For insulin, glucagon, and somatostatin immunoreactivity, three parameters were determined: relative immunoreactive islet area, percentage of immunoreactive cells, and immunoreactive cell density. Quantitative assessment was performed in ImageJ after color deconvolution using the hematoxylin-DAB setting. The DAB-separated channel was used for segmentation of hormone-positive area. For each marker, the threshold defining DAB positivity was established before group decoding on representative images covering the observed staining range and was then applied unchanged to all images analyzed for that marker. Thresholding was performed only within the manually delineated islet ROI. The same image-acquisition settings, thresholding procedure, and inclusion criteria were applied to all experimental groups.

The total area of each pancreatic islet was measured and expressed in µm^2^. The area occupied by the positive DAB signal within the manually delineated islet ROI was then measured and expressed as a percentage of the total islet area according to the formula:

$$IR\;area\;\left(\%\right)=\;\left(\frac{immunoreactive\;area}{total\;islet\;area}\right)\times 100$$

In the same islets, the total number of cells and the number of hormone-immunoreactive cells were counted manually. Cells were classified as immunoreactive when a brown cytoplasmic DAB signal was clearly distinguishable from the local background and was associated with an identifiable cell body within the islet. Weakly positive cells were included only when the signal was discrete, cytoplasmic, and above the surrounding background. Diffuse background staining, staining precipitates, poorly defined cellular profiles, and equivocal signals were not counted as positive. The same criteria were applied for insulin-, glucagon-, and somatostatin-positive cells in all groups. The percentage of immunoreactive cells was calculated according to the formula:

$$IR\;cells\;\left(\%\right)=\;\left(\frac{number\;of\;immunoreactive\;cells}{total\;number\;of\;cells\;within\;the\;islet}\right)\times \;100$$

Immunoreactive cell density was calculated by normalizing the number of immunoreactive cells to the total islet area and expressed as cells per 1000 µm^2^:

$$IR\;cell\;density\;=\;\left(\frac{number\;of\;immunoreactive\;cells}{total\;islet\;area}\right)\times \;1000$$

For PCNA immunoreactivity, positive cells were identified on the basis of nuclear DAB staining within whole pancreatic islet profiles. A nucleus was classified as PCNA-positive when brown nuclear staining was clearly visible above the hematoxylin counterstain and local background. The total number of islet nuclei and the number of PCNA-positive nuclei were counted within each islet ROI. The result was expressed as islet PCNA-positive cell percentage. Because PCNA staining was not combined with insulin, glucagon, or somatostatin immunostaining, the endocrine-cell identity of PCNA-positive cells was not determined:

$$PCNA-positive\;cells\;\left(\%\right)=\;\left(\frac{number\;of\;PCNA\;positive\;cells}{total\;number\;of\;cells\;within\;the\;islet}\right)\times \;100$$

For cleaved caspase-3 immunoreactivity, optical density was measured on the DAB-separated channel after color deconvolution [23]. Measurements were performed without threshold-based segmentation, as the aim was to determine the mean DAB optical density within the entire manually delineated islet ROI. Local background intensity was determined using background ROIs selected from non-immunoreactive areas of the same image whenever available, or from the nearest comparable field on the same section when necessary. Background ROIs were selected according to the same criteria for all samples and were placed in areas free of specific DAB signal, tissue folds, section edges, vessels, lumina, staining precipitates, and other artifacts. Weakly immunoreactive areas were not used for background measurement. Mean optical density was calculated according to the formula:

$$OD\;=\;{log}_{10}(\frac{I_{0}}{I}),$$

where I_0_ represents the mean gray value of the local background/reference area measured on the DAB-separated channel, and I represents the mean gray value of the analyzed pancreatic islet ROI measured on the same channel. Optical density was treated as a dimensionless value and reported in arbitrary units.

For each animal, values obtained from the 10 analyzed pancreatic islets were averaged to obtain one representative value per animal for statistical analysis. Before image analysis, all samples were coded, and the observer performing the measurements was blinded to both treatment group and sex.

Islet profiles were analyzed only when the complete islet outline was visible and could be included within the analyzed image field. Large islet profiles extending beyond the field of view were not analyzed partially; when such profiles were encountered, the next eligible complete islet profile was selected according to the same systematic sampling sequence. Islet profiles excluded because of folds, tears, section-edge artifacts, staining precipitates, excessive background staining, or incomplete visualization were replaced by the next eligible profile using the same predefined selection procedure.

### 2.6. Statistical Analysis

Statistical analyses were performed using GraphPad Prism version 11.0.1 (GraphPad Software, Boston, MA, USA). The individual animal was the experimental unit (n = 8 per sex/treatment group). For each immunohistochemical endpoint, measurements from 10 pancreatic islets were averaged within each animal to obtain one value for analysis; individual islets were treated as subsamples and not as independent observations.

Each outcome was analyzed by ordinary two-way ANOVA for a 2 × 2 factorial design, with sex (male or female) and treatment (control or irisin) as between-subject factors and with the sex × treatment interaction included in the model. The interaction term was used to evaluate whether the effect of irisin differed between males and females.

Where pairwise comparisons were required to characterize differences among experimental groups, the post hoc comparison family was defined separately for each endpoint. For each biochemical and immunohistochemical endpoint, four predefined pairwise comparisons were considered: control vs. irisin-treated males, control vs. irisin-treated females, control males vs. control females, and irisin-treated males vs. irisin-treated females. Diagonal comparisons between unrelated sex/treatment combinations were not included. Multiplicity was controlled separately within each endpoint by applying the sequential Holm–Bonferroni procedure to these four post hoc *p*-values. The *p*-values reported in Section 3 for pairwise post hoc comparisons and used for figure annotations are Holm–Bonferroni-adjusted *p*-values. All tests were two-sided, and *p* < 0.05 was considered statistically significant. Data are presented as arithmetic mean ± SEM.

All 32 animals completed the experiment. No animals, tissue samples, or immunohistochemical reactions were lost or excluded from the planned analyses. All biochemical and immunohistochemical endpoints were analyzed in all animals, with n = 8 animals per sex/treatment group. For each immunohistochemical endpoint, 10 pancreatic islet profiles were analyzed per animal and averaged to obtain one animal-level value for statistical analysis.

## 3. Results

### 3.1. Serum Biochemical Parameters

**Figure 1:**
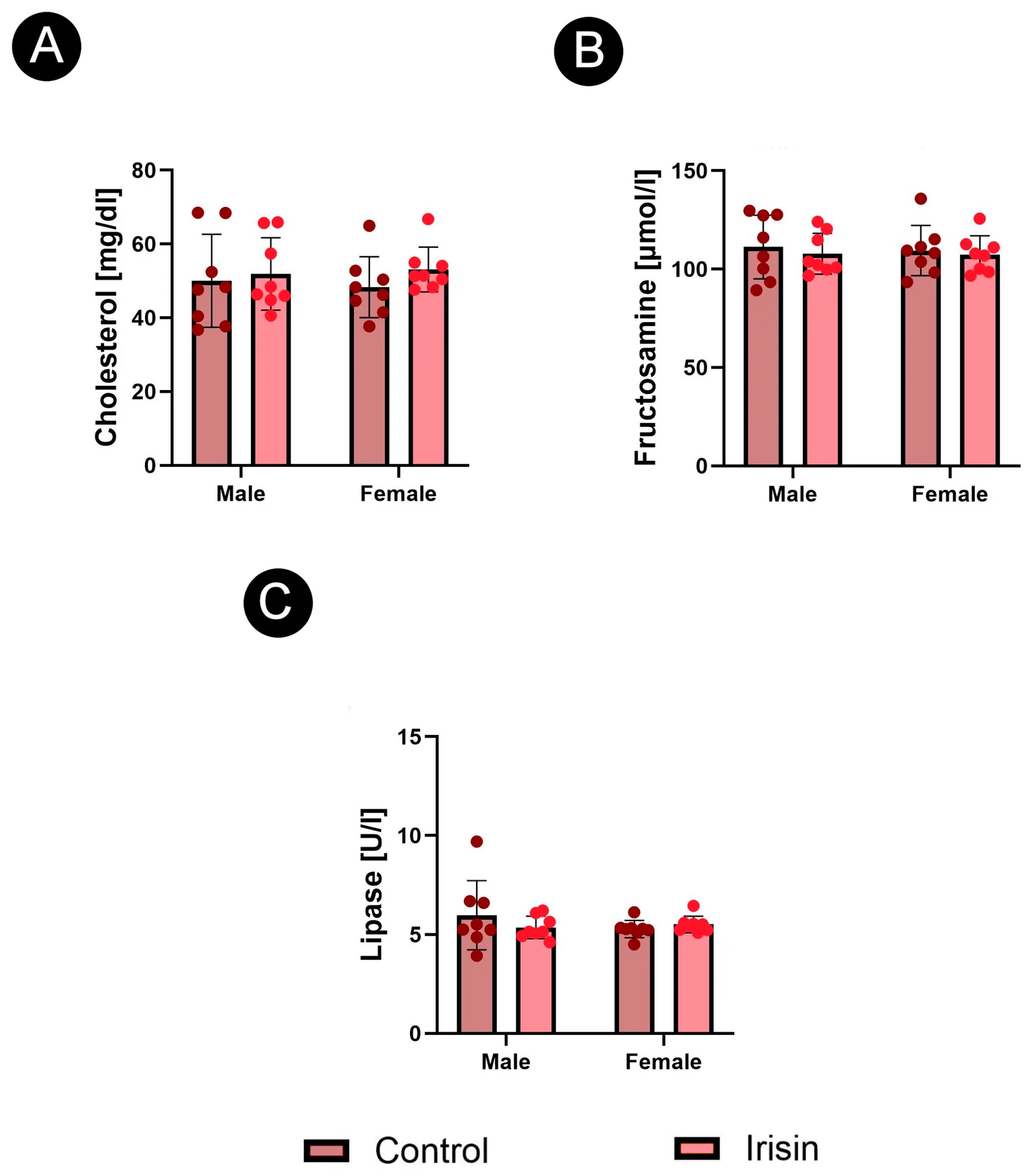
Serum biochemical parameters in male and female Wistar rats after exogenous irisin administration: (**A**) serum cholesterol concentration, (**B**) serum fructosamine concentration, and (**C**) serum lipase activity. Data are presented as mean ± SEM (n = 8 animals per sex/treatment group) and were analyzed by ordinary two-way ANOVA.

Serum cholesterol, fructosamine, and lipase were assessed as supportive biochemical endpoints to determine whether exogenous irisin administration was accompanied by major systemic metabolic changes or biochemical evidence of pancreatic injury. Irisin treatment did not significantly affect any of these parameters in either males or females. No significant sex-related differences were detected within the control or irisin-treated groups. No significant sex × irisin interaction was detected for serum cholesterol, fructosamine, or lipase. Thus, the pancreatic endocrine immunophenotypic changes were not accompanied by significant alterations in serum cholesterol, fructosamine, or lipase activity under the applied experimental conditions (Figure 1A–C).

### 3.2. Analysis of Pancreatic α-Cells

A significant sex × irisin interaction was detected for glucagon-IR area (*p* = 0.001) and the percentage of α-IR cells (*p* = 0.016).

**Figure 2:**
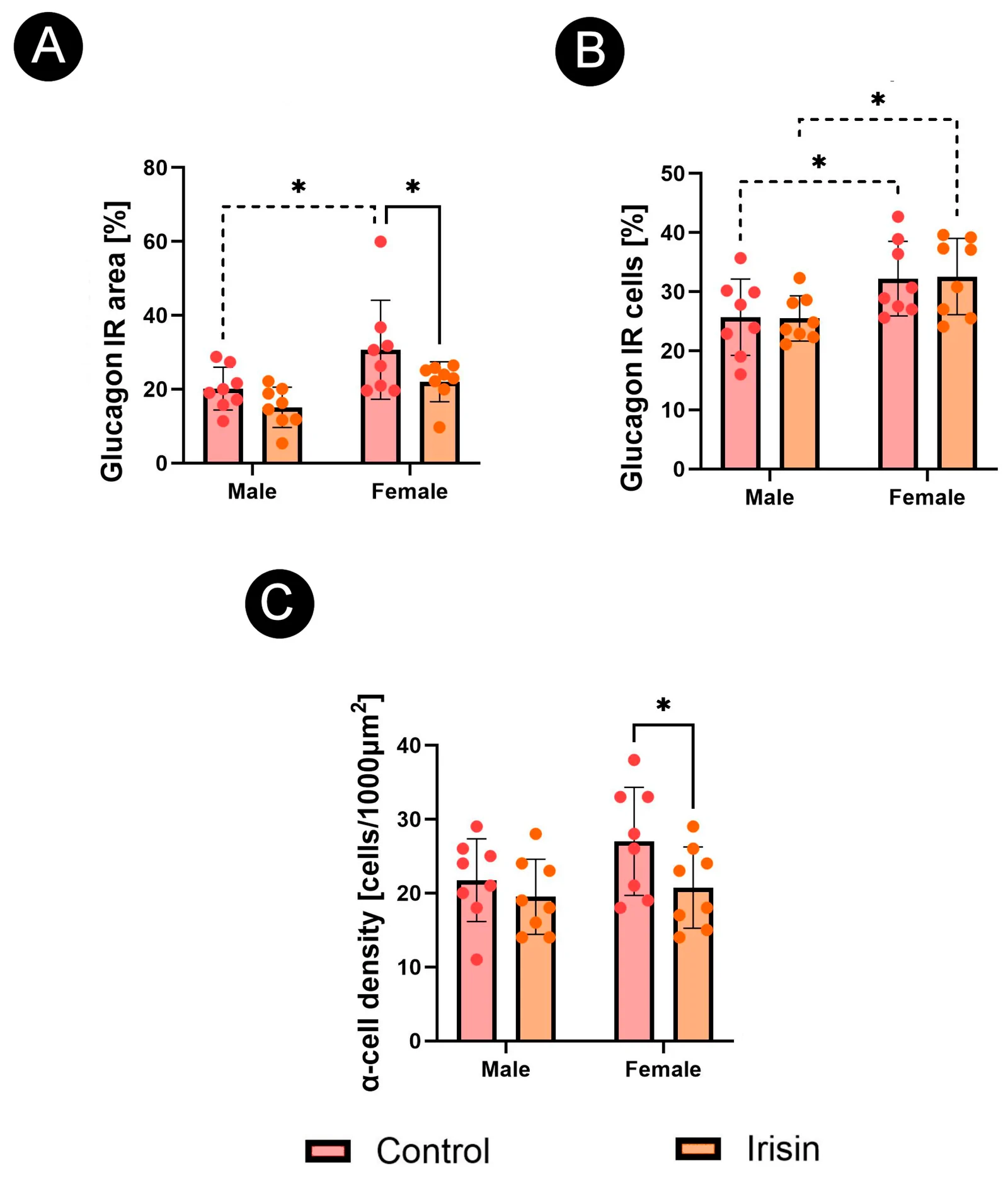
Effect of exogenous irisin on pancreatic α-cell immunoreactivity in male and female Wistar rats: (**A**) relative immunoreactive islet area, (**B**) immunoreactive cell percentage, and (**C**) cell density. Data are presented as mean ± SEM (n = 8 animals per sex/treatment group) and were analyzed by ordinary two-way ANOVA. Brackets denote statistically significant post hoc comparisons; solid brackets compare control and irisin treatment within a sex, and dashed brackets compare sexes within a treatment group. * *p* < 0.05.

Pancreatic α-cell IR was assessed on the basis of glucagon-positive staining. Irisin administration decreased the glucagon-IR area in females (*p* = 0.044), whereas no significant treatment-related change was detected in males. In the control groups, glucagon-IR area was higher in females than in males (*p* = 0.016; Figure 2A).

The percentage of glucagon-IR cells was not significantly affected by irisin administration in either sex. However, this parameter remained higher in females than in males both under control conditions (*p* = 0.034) and after irisin treatment (*p* = 0.022; Figure 2B).

**Figure 3:**
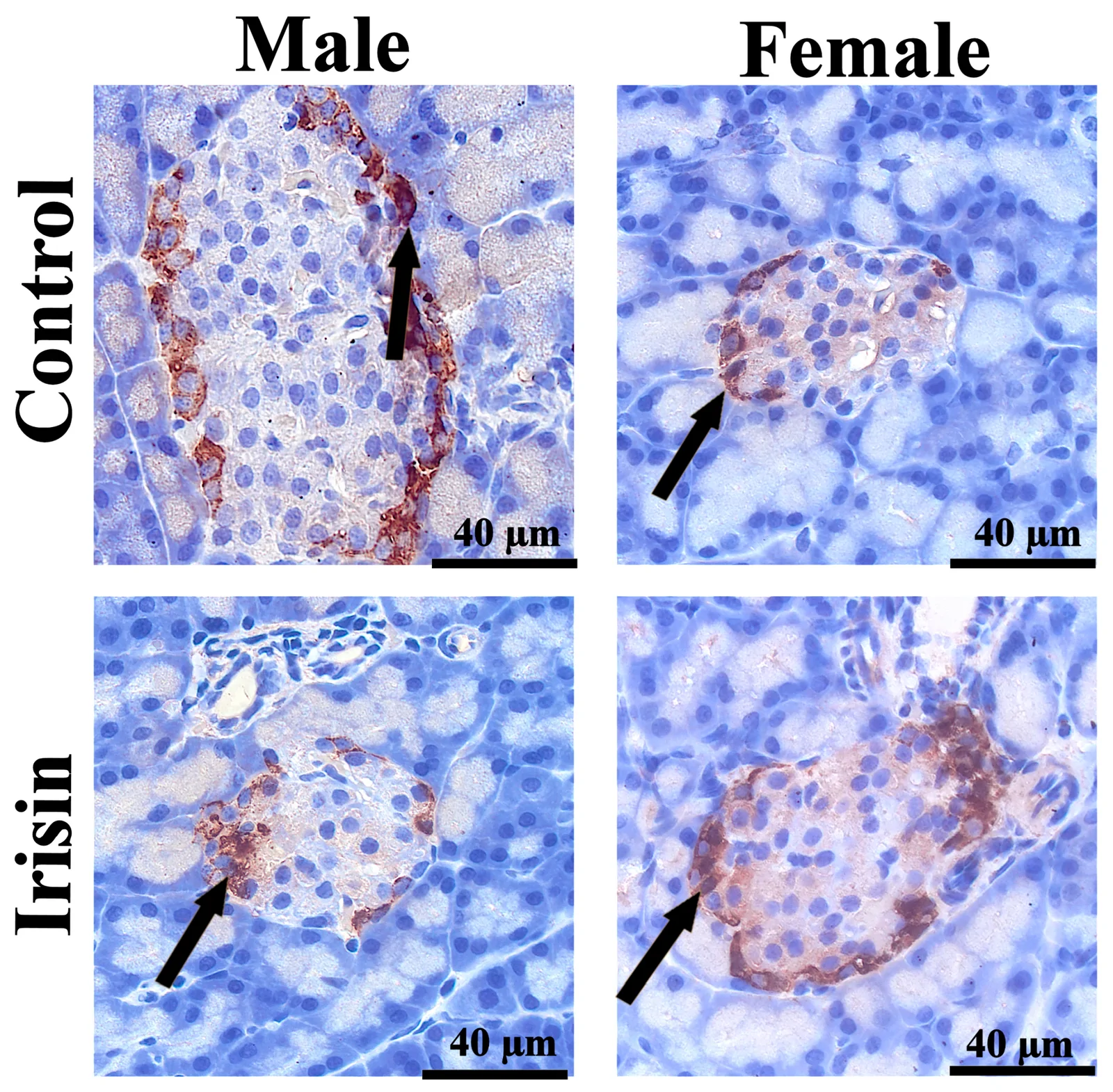
Representative photomicrographs of immunohistochemical reactions for glucagon in pancreatic islets of male and female control and irisin-treated Wistar rats. Black arrows indicate glucagon-immunoreactive cells. Scale bars = 40 µm.

Glucagon-IR cell density decreased in females after irisin administration (*p* = 0.044), while no significant treatment-related change was observed in males (Figure 2C). Representative immunoreactivity is shown in Figure 3.

### 3.3. Analysis of Pancreatic β-Cells

A significant sex × irisin interaction was detected for β-IR area (*p* = 0.003), the percentage of insulin-IR cells (*p* = 0.018), and insulin-IR cell density (*p* = 0.033).

**Figure 4:**
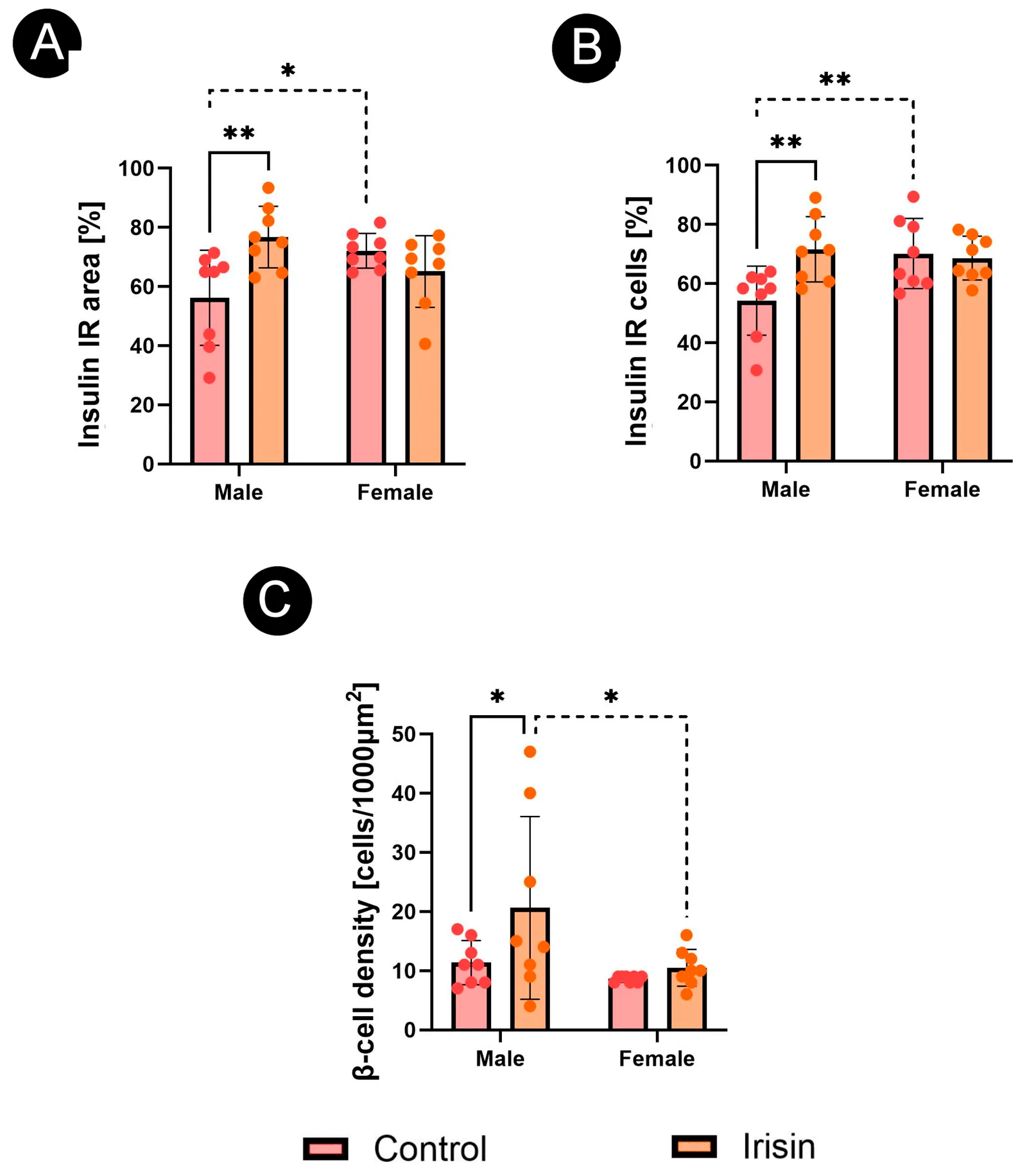
Effect of exogenous irisin on pancreatic β-cell immunoreactivity in male and female Wistar rats: (**A**) relative immunoreactive islet area, (**B**) immunoreactive cell percentage, and (**C**) cell density. Data are presented as mean ± SEM (n = 8 animals per sex/treatment group) and were analyzed by ordinary two-way ANOVA. Brackets denote statistically significant post hoc comparisons; solid brackets compare control and irisin treatment within a sex, and dashed brackets compare sexes within a treatment group. * *p* < 0.05; ** *p* < 0.01.

Pancreatic β-cell IR was assessed on the basis of insulin-positive staining. In males, irisin administration increased the insulin-IR area compared with the control group (*p* = 0.002). No treatment-related change in insulin-IR area was detected in females. In the control groups, insulin-IR area was lower in males than in females (*p* = 0.011; Figure 4A), whereas this sex difference was no longer evident after irisin administration.

A similar pattern was observed for the percentage of insulin-IR cells. Irisin increased the proportion of insulin-IR cells in males (*p* = 0.003), while no significant change was observed in females. Under control conditions, females showed a higher percentage of insulin-IR cells than males (*p* = 0.006). After irisin treatment, this difference was not significant (Figure 4B).

**Figure 5:**
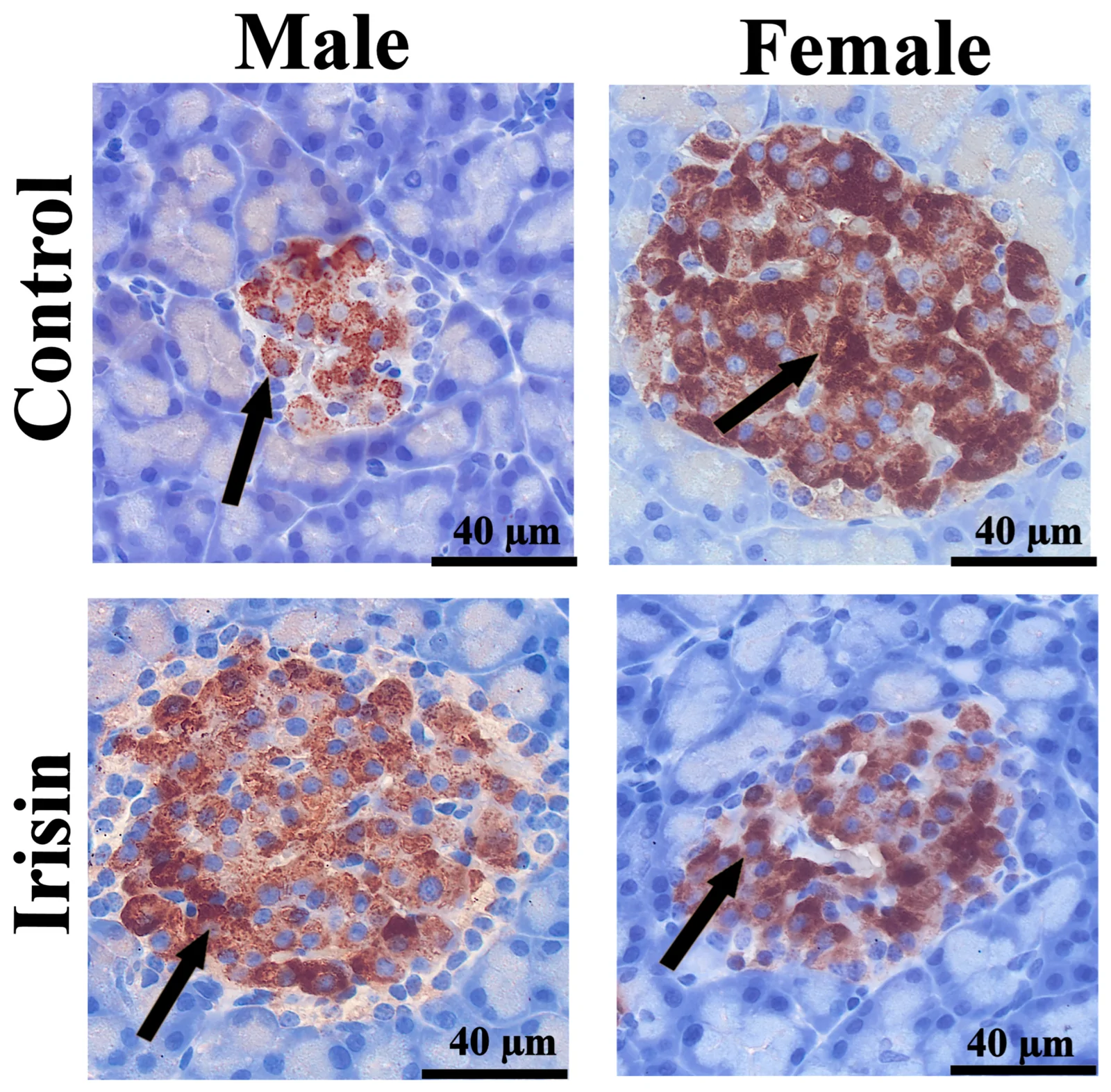
Representative photomicrographs of immunohistochemical reactions for insulin in pancreatic islets of male and female control and irisin-treated Wistar rats. Black arrows indicate insulin-immunoreactive cells. Scale bars = 40 µm.

Insulin-IR cell density also increased in males following irisin administration (*p* = 0.030). In the irisin-treated groups, insulin-IR cell density was higher in males than in females (*p* = 0.019; Figure 4C). Representative immunoreactivity is shown in Figure 5.

### 3.4. Analysis of Pancreatic δ-Cells

A significant sex × irisin interaction was detected for δ-cell IR area (*p* = 0.006) and somatostatin-IR cell density (*p* = 0.003).

**Figure 6:**
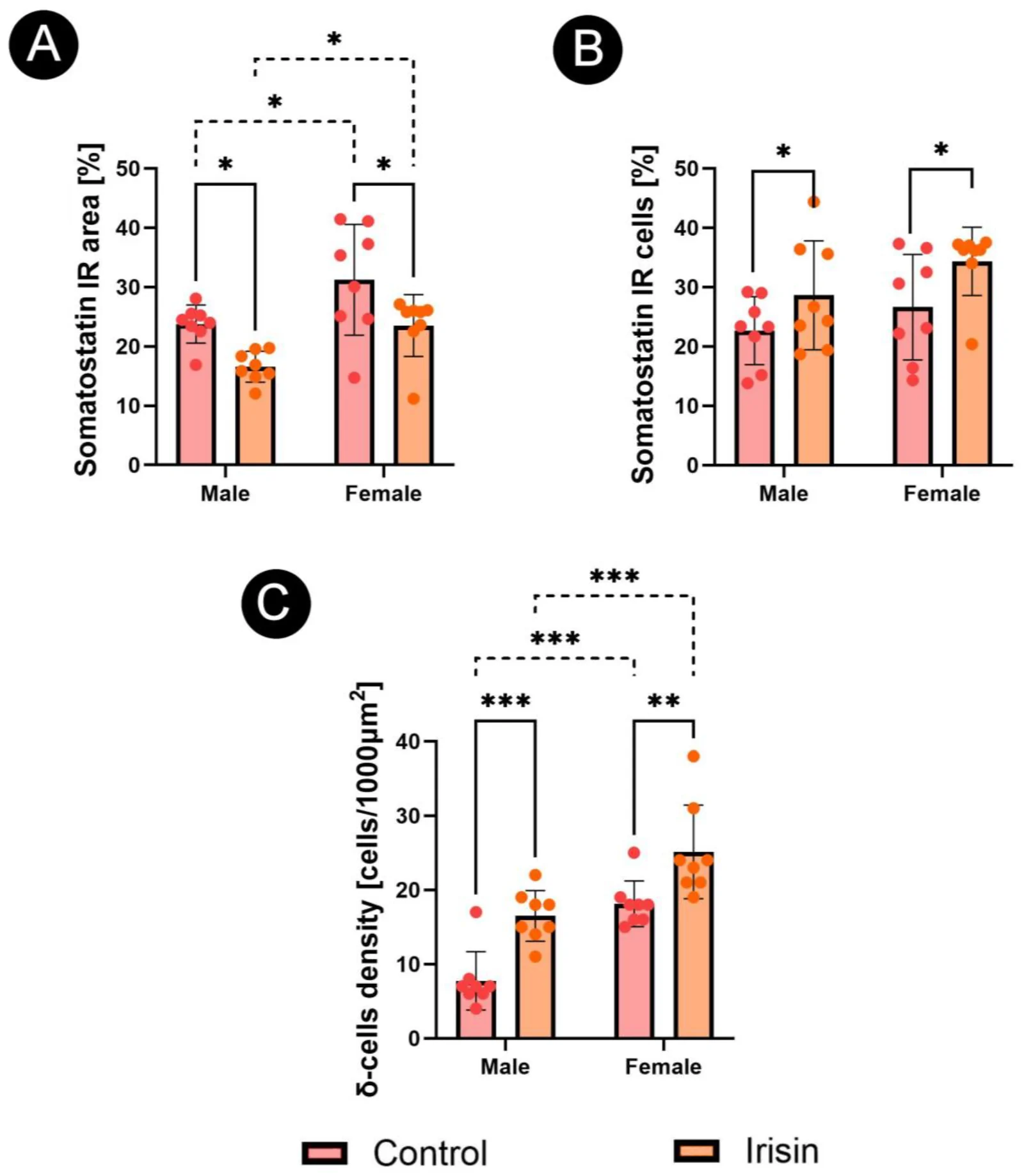
Effect of exogenous irisin on pancreatic δ-cell immunoreactivity in male and female Wistar rats: (**A**) relative immunoreactive islet area, (**B**) immunoreactive cell percentage, and (**C**) cell density. Data are presented as mean ± SEM (n = 8 animals per sex/treatment group) and were analyzed by ordinary two-way ANOVA. Brackets denote statistically significant post hoc comparisons; solid brackets compare control and irisin treatment within a sex, and dashed brackets compare sexes within a treatment group. * *p* < 0.05; ** *p* < 0.01; *** *p* < 0.001.

Pancreatic δ-cell IR was assessed on the basis of somatostatin-positive staining. Irisin administration decreased the somatostatin-IR area in both males (*p* = 0.018) and females (*p* = 0.012). In both treatment conditions, somatostatin-IR area was higher in females than in males, with significant sex-related differences observed in the control group (*p* = 0.015) and in the irisin-treated group (*p* = 0.022; Figure 6A).

**Figure 7:**
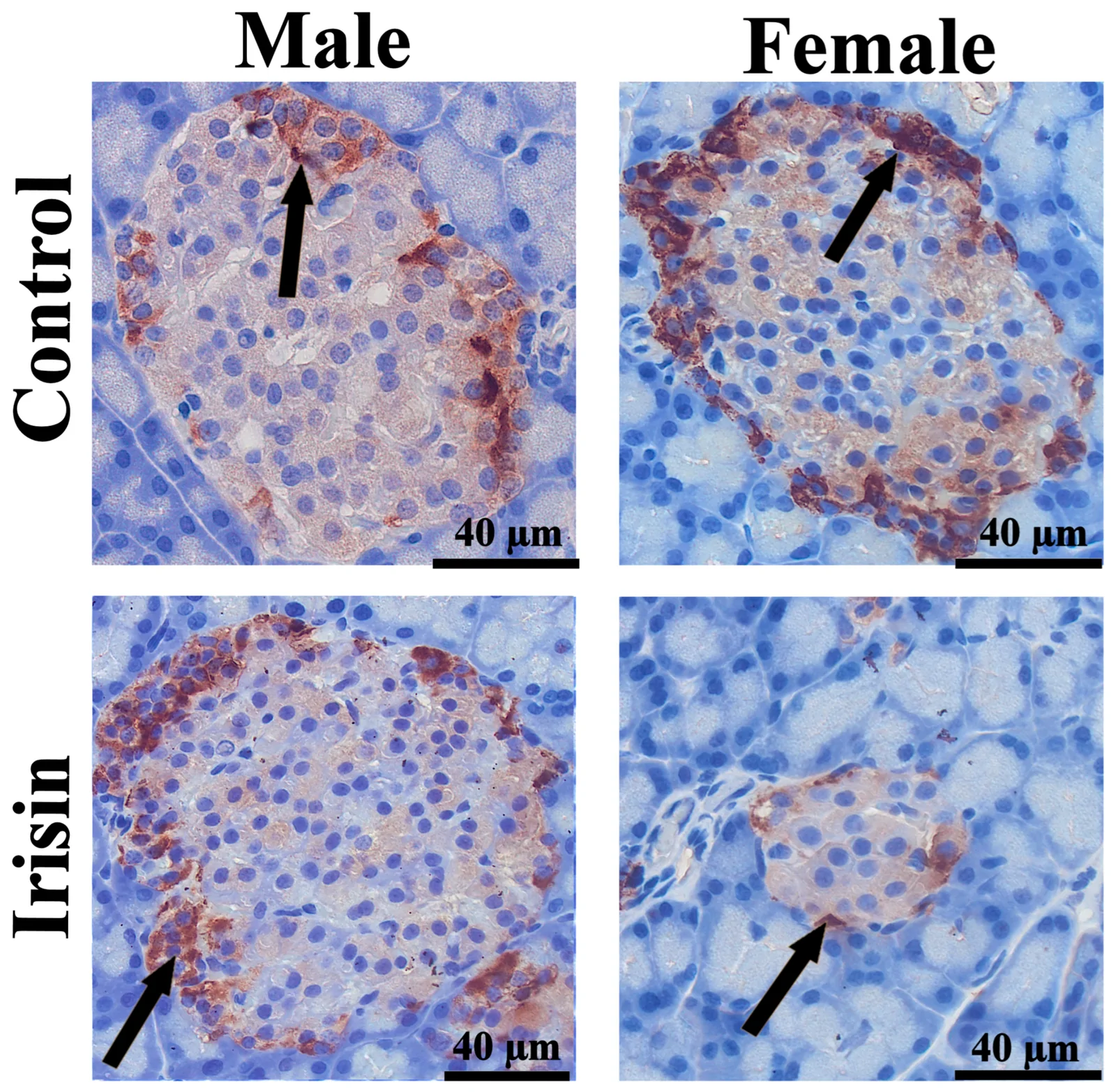
Representative photomicrographs of immunohistochemical reactions for somatostatin in pancreatic islets of male and female control and irisin-treated Wistar rats. Black arrows indicate somatostatin-immunoreactive cells. Scale bars = 40 µm.

Despite the reduction in somatostatin-IR area, the percentage of somatostatin-IR cells increased after irisin administration in both males (*p* = 0.012) and females (*p* = 0.049; Figure 6B). Somatostatin-IR cell density showed a similar treatment-related increase, with higher values detected after irisin administration in males (*p* < 0.001) and females (*p* = 0.003; Figure 6C). In contrast to the somatostatin-IR area, cell density was higher in females than in males in both the control and irisin-treated groups (both *p* < 0.001; Figure 6C). Representative immunoreactivity is shown in Figure 7.

### 3.5. Analysis of Proliferation and Apoptosis

**Figure 8:**
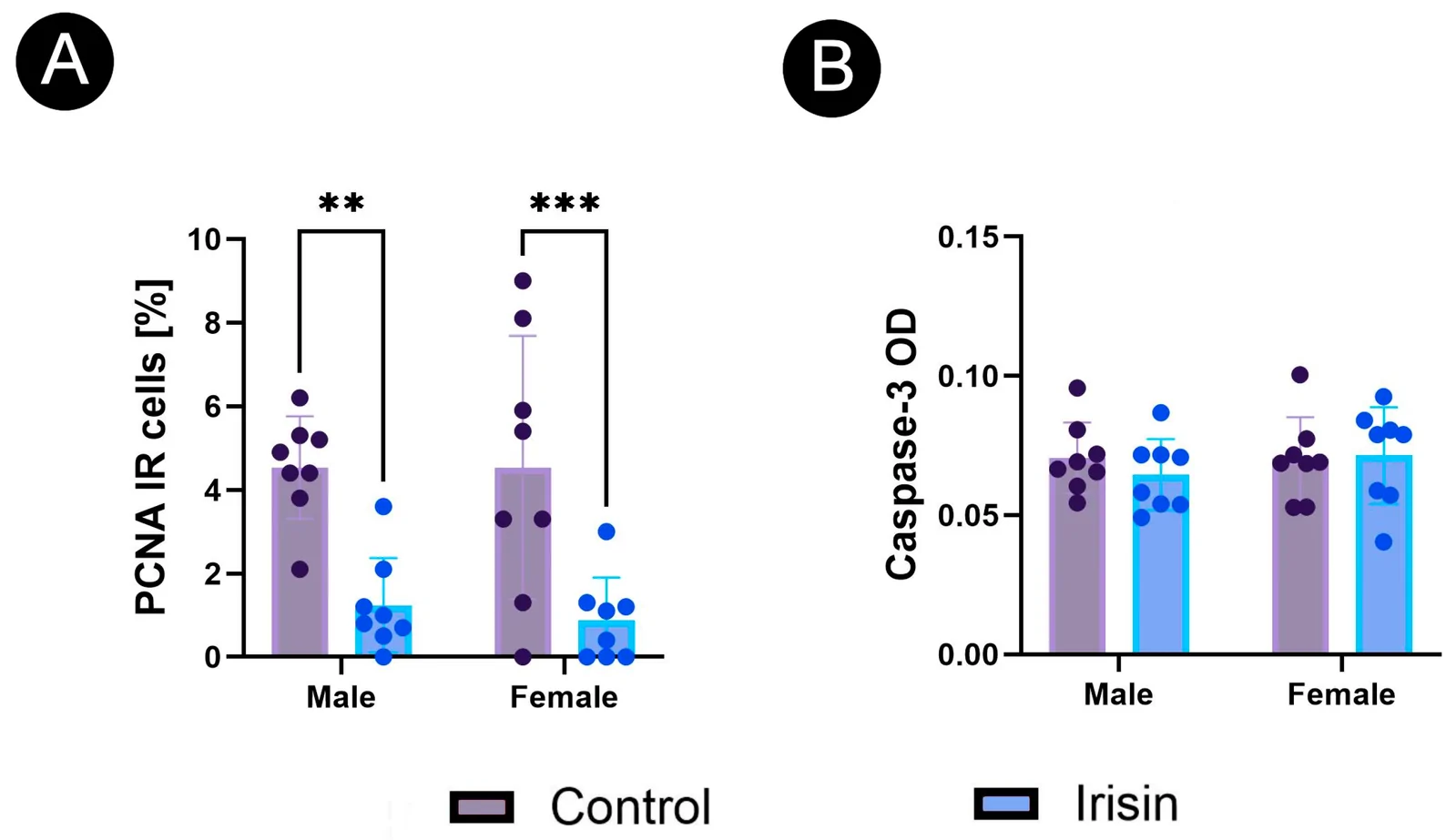
Effect of exogenous recombinant FNDC5/irisin on islet PCNA and cleaved caspase-3 immunoreactivity in male and female Wistar rats: (**A**) islet PCNA-positive cell percentage, (**B**) islet cleaved caspase-3 optical density. Data are presented as mean ± SEM (n = 8 animals per sex/treatment group) and were analyzed by ordinary two-way ANOVA. Brackets denote statistically significant post hoc comparisons; solid brackets compare control and irisin treatment within a sex. ** *p* < 0.01; *** *p* < 0.001.

PCNA immunoreactivity was assessed as the percentage of PCNA-positive cells within whole pancreatic islet profiles. Recombinant FNDC5/irisin administration decreased the islet PCNA-positive cell percentage in both males (*p* = 0.001) and females (*p* < 0.001; Figure 8A).

Cleaved caspase-3 immunoreactivity was assessed as optical density within whole pancreatic islet profiles. Islet cleaved caspase-3 immunoreactivity, assessed as optical density, was not significantly altered by recombinant FNDC5/irisin administration in either males or females (Figure 8B).

## 4. Discussion

The present study indicates that repeated intermittent administration of recombinant FNDC5/irisin during the post-weaning period is associated with marker-, sex-, and endocrine-compartment-specific changes in pancreatic islet immunophenotype. Rather than suggesting a uniform trophic effect on the endocrine pancreas, the pattern of responses points to selective changes in hormone-related immunoreactivity within developing islets. Because glucose tolerance, circulating endocrine hormones, islet hormone release, pancreatic FNDC5/irisin exposure, and direct apoptosis assays were not assessed, these findings should be interpreted as descriptive tissue-level changes rather than as direct evidence of altered islet endocrine function or apoptosis.

This study should be interpreted as a descriptive immunohistochemical assessment of pancreatic islet phenotype rather than as a functional analysis of islet hormone secretion. Immunoreactive area, immunoreactive cell percentage, and immunoreactive cell density provide information on the hormone-positive tissue compartment within pancreatic islets, but they do not directly measure insulin, glucagon, or somatostatin release. Accordingly, the increased insulin immunoreactivity observed in males may indicate an increased insulin-positive islet compartment or altered stored insulin immunoreactivity, but it cannot be interpreted as evidence of increased insulin secretion or improved glucose tolerance. Similarly, the reduced glucagon immunoreactivity observed in females may reflect a change in the glucagon-positive compartment or glucagon immunostaining profile, but it does not establish reduced glucagon secretion. The somatostatin findings should also be interpreted at the tissue-immunophenotype level, because somatostatin-positive area and somatostatin-positive cell number do not directly indicate δ-cell secretory activity. Although unchanged serum fructosamine suggests that these tissue-level changes were not accompanied by a detectable alteration in short-term integrated glycemic status, fructosamine cannot replace intraperitoneal glucose tolerance testing, insulin tolerance testing, fasting insulin/glucagon measurements, or ex vivo islet secretion assays.

The serum biochemical findings are important because they provide a safety and metabolic context for the tissue-level observations. Serum lipase was included as a supportive marker of pancreatic injury. In rat models of exocrine pancreatic injury, lipase and amylase can increase with pancreatic damage, although their magnitude does not always parallel histological severity and their sensitivity is limited [24]. In the present study, unchanged lipase activity suggests that the irisin protocol was not associated with biochemical evidence of overt pancreatic injury. Fructosamine was used as a short-term glycemic marker. Since fructosamine reflects glycated circulating proteins over a shorter period than HbA1c, it may detect recent changes in glycemic status, although it is influenced by serum protein turnover and does not replace functional tests of glucose homeostasis [25]. The lack of a fructosamine change indicates that the pancreatic immunohistochemical changes were not accompanied by a detectable shift in integrated glycemic status. Unchanged cholesterol further suggests that the endocrine-cell changes did not occur together with a broad alteration in circulating cholesterol, which is relevant because β-cell cholesterol handling can influence insulin secretion and glucose homeostasis [26].

The male-specific increase in insulin immunoreactivity is consistent with earlier evidence that irisin can act on pancreatic β-cells. Natalicchio et al. reported that irisin promoted β-cell survival and insulin secretion under saturated fatty acid exposure, while Liu et al. showed that irisin increased proliferation and reduced apoptosis in INS-1 cells and in a diabetic rat model [7,8]. Zhang et al. also demonstrated protective effects of irisin against glucolipotoxic β-cell dysfunction through AMPK-associated mechanisms [9]. These studies support the idea that β-cells are responsive to irisin. However, the present model differs from most previous work because the rats were healthy and post-weaning, rather than diabetic or metabolically stressed. Therefore, increased insulin-IR area, insulin-IR cell percentage, and insulin-IR cell density should be interpreted as changes in insulin-positive tissue phenotype, not as direct evidence of increased insulin secretion or improved glucose tolerance.

The sex-specific nature of the β-cell response is also consistent with the broader literature on endocrine pancreas biology. Earlier rat work showed that age and sex affect endocrine pancreas structure and insulin secretory function [27]. More recent reviews and transcriptomic studies also support the view that biological sex influences islet-cell biology, stress responses, and endocrine-cell gene programs [28,29]. In the present study, females had higher insulin-IR area and insulin-IR cell percentage than males under control conditions, whereas irisin increased these parameters mainly in males. This suggests that irisin did not act on a neutral baseline. Instead, its effect may have depended on pre-existing sex-related differences in the developing endocrine pancreas.

The α-cell response differed from the β-cell response. Irisin decreased glucagon-IR area and glucagon-IR cell density in females, while the percentage of glucagon-IR cells was not significantly altered by treatment. This suggests that irisin did not simply reduce the proportion of α-cells, but may have affected glucagon-positive area, α-cell profile, or local α-cell distribution. Glucagon secretion is regulated by α-cell mechanisms and by paracrine signals from neighboring β- and δ-cells, including insulin, zinc, and somatostatin [30,31]. The female-specific reduction in glucagon immunoreactivity may therefore reflect an adjustment within the islet network rather than an isolated α-cell effect. A useful comparison comes from female mice fed a high-fat diet, in which α-cells showed altered glucagon content and α-cell area during a compensatory metabolic state [32]. That model differs from the present study because it involved obesity and insulin resistance, but it supports the broader point that α-cell immunophenotype can change without a simple increase or decrease in α-cell percentage.

The δ-cell response was more complex. Somatostatin-IR area decreased in both sexes, whereas somatostatin-IR cell percentage and density increased. This combination should not be reduced to a simple statement that somatostatin-positive cells increased or decreased. A lower immunoreactive area together with higher cell density may reflect smaller somatostatin-positive profiles, altered distribution of δ-cells within islets, or a change in the relationship between hormone-positive area and cell number. δ-cells are important paracrine regulators of both insulin and glucagon secretion [14]. Their morphology also supports broad local communication within the islet, since δ-cells can extend processes that contact multiple endocrine cells [33]. In addition, somatostatin secretion is regulated by insulin- and glucagon-related paracrine signaling [34]. These findings make it plausible that the δ-cell changes observed here are part of a broader reorganization of intra-islet signaling after irisin administration.

The developmental stage of the animals is central to the interpretation of these results. Rodent β-cells are not fully mature at birth, and weaning is associated with major functional and molecular changes. Stolovich-Rain et al. showed that weaning triggers a distinct β-cell maturation step, increasing the secretory and mitogenic response to glucose [35]. Jacovetti et al. further showed that postnatal β-cell maturation is associated with islet-specific microRNA changes induced by the nutritional transition at weaning [36]. This is important because the present rats were treated from 3 to 7 weeks of age, a period in which endocrine pancreas maturation is still ongoing. Thus, the response to irisin in this study should not be expected to mirror adult diabetic or pancreatitis models.

The decrease in PCNA-IR cell percentage in both sexes contrasts with previous findings from stressed β-cell models, in which irisin increased β-cell proliferation and reduced apoptosis under high-glucose or glucolipotoxic conditions [8,9]. In the present healthy post-weaning rats, however, PCNA immunoreactivity decreased while cleaved caspase-3 OD remained unchanged. This pattern does not indicate a detectable increase in cleaved caspase-3 immunoreactivity under the applied method. Rather, it may indicate a shift toward a less proliferative state during pancreatic maturation. This interpretation is consistent with evidence that β-cell replication is associated with a more immature state, whereas functionally specialized β-cells generally show lower proliferative activity [37]. Reviews of β-cell maturation also emphasize that postnatal β-cell development involves coordinated changes in cell-cycle activity, metabolism, and glucose responsiveness [38]. The lack of change in cleaved caspase-3 OD does not contradict previous reports of irisin-mediated β-cell protection, because anti-apoptotic effects are most evident in models in which apoptosis is increased by metabolic or oxidative stress [8,9,10]. Thus, reduced PCNA in this model may reflect altered maturation dynamics rather than toxicity, while unchanged cleaved caspase-3 OD is consistent with the absence of overt pancreatic injury. However, PCNA and cleaved caspase-3 were not co-localized with endocrine-cell markers in the present study. Consequently, the observed changes cannot be assigned specifically to β-, α-, or δ-cells or interpreted as direct evidence of altered endocrine-cell proliferation or apoptosis. In the present healthy post-weaning rats, however, PCNA immunoreactivity was assessed within whole pancreatic islet profiles and was not co-localized with endocrine-cell markers. Therefore, the decrease in PCNA-positive cells cannot be assigned specifically to β-, α-, or δ-cells. This finding should be interpreted as a reduction in islet-level PCNA-positive cell percentage rather than direct evidence of reduced endocrine-cell proliferation.

Although the present study did not examine exercise, the findings can be considered in the context of the PGC-1α/FNDC5/irisin axis. In physiological conditions, irisin is linked to skeletal-muscle PGC-1α activity and exercise-associated signaling, whereas the present design bypassed this upstream regulation by administering recombinant irisin directly. Thus, the study should not be interpreted as an exercise model, but rather as an assessment of one endocrine mediator associated with exercise-related signaling. Because mitochondrial metabolism is central to β-cell stimulus–secretion coupling and postnatal endocrine maturation, mitochondrial remodeling may represent a plausible mechanism through which irisin could influence pancreatic endocrine-cell phenotype. However, physical activity, PGC-1α expression, mitochondrial biogenesis, and mitochondrial function were not assessed, and therefore, this mechanism remains hypothetical.

Several limitations should be considered. First, IR area, IR cell percentage, cell density, and optical density describe tissue immunophenotype, but they do not directly measure hormone secretion. Serum fructosamine provides only indirect information on intermediate-term glycemic status and cannot replace glucose tolerance tests, insulin tolerance tests, or direct hormone assays. In addition, circulating insulin, glucagon, somatostatin, and irisin/FNDC5 concentrations were not measured. Second, PCNA and cleaved caspase-3 were evaluated without co-localization with insulin, glucagon, or somatostatin. These measurements therefore reflected IR within pancreatic islets as a whole and could not identify β-, α-, or δ-cell-specific changes in proliferation-associated or cleaved caspase-3-associated immunoreactivity. Moreover, no biological positive-control group known to induce β-cell proliferation or islet apoptosis was included. Such a group would have strengthened the interpretation of PCNA and cleaved caspase-3 staining and the dynamic range of these IHC endpoints. Third, the analysis was based on two-dimensional islet profiles. Although mean sampled islet profile area was evaluated as a sampling-control parameter, the study did not include stereological assessment of total islet size distribution, islet mass, or endocrine-cell mass. Therefore, residual bias related to islet size, sectioning plane, or endocrine-cell distribution within islets cannot be fully excluded. Finally, a limitation of the study concerns the recombinant FNDC5/irisin preparation used for treatment. Although the administered protein corresponded to the reported mature/secreted irisin region of rat FNDC5 (aa 29–140), it was produced in *E. coli* and contained an N-terminal 6 × His tag. The specific lot used in the present study was not independently tested by the authors in a cell-based bioactivity assay. Recombinant irisin has been reported to have a short in vivo half-life; therefore, a single terminal blood measurement, particularly one week after the final injection, would not be expected to confirm transient exposure after dosing. Direct confirmation would require a separate pharmacokinetic analysis with serial blood sampling shortly after administration. Consequently, the observed pancreatic immunohistochemical differences are consistent with biological activity of the administered recombinant FNDC5/irisin preparation, but they cannot by themselves confirm systemic exposure to biologically active circulating irisin.

## 5. Conclusions

In conclusion, administration of exogenous recombinant FNDC5/irisin was associated with changes in pancreatic islet immunophenotype in post-weaning Wistar rats, without significant changes in serum cholesterol, fructosamine, or lipase and without increased islet cleaved caspase-3 optical density. Males showed increased insulin immunoreactivity, females showed reduced glucagon immunoreactivity, and both sexes showed changes in somatostatin immunoreactivity and reduced islet PCNA-positive cell percentage. Because glucose tolerance, circulating insulin/glucagon concentrations, and islet hormone-release assays were not assessed, these findings should be interpreted as descriptive tissue-level changes and cannot establish altered islet endocrine function or glucose homeostasis.

## Figures and Tables

**Table 1:** Primary and secondary antibodies used in the study.

Target Antigen	Antibody Type	Host Species	Manufacturer (Location)	Catalog Number	Dilution
Secondary detection system Anti-mouse/rabbit	Polyclonal, HRP- conjugated, secondary	Goat	ImmunoLogic (The Netherlands)	DPVB-HRP	RTU ^1^
Glucagon	Monoclonal, primary	Mouse	ThermoFisher (Waltham, MA, USA)	14-9743-82	1:200
Insulin	Monoclonal, primary	Mouse	ThermoFisher	MA5-12037	1:200
Somatostatin	Polyclonal, primary	Rabbit	ThermoFisher	PA585759	1:500
Proliferating-cell nuclear antigen (PCNA)	Polyclonal, primary	Rabbit	Affinity Biosciences (Cincinnati, OH, USA)	AF0239	1:150
Cleaved caspase-3	Monoclonal, primary	Mouse	Proteintech (Rosemont, IL, USA)	68773-1-IG	1:2000

^1^ Ready to use.

## Data Availability

The raw data supporting the conclusions of this article will be made available by the authors on request.

## Abbreviations

The following abbreviations are used in this manuscript:

FNDC5	Fibronectin type III domain-containing protein 5
PGC-1α	Peroxisome-proliferator-activated receptor gamma coactivator 1-alpha
PCNA	Proliferating-cell nuclear antigen

## References

[1] Chen W., Wang L., You W., Shan T. (2021). Myokines Mediate the Cross Talk between Skeletal Muscle and Other Organs. J. Cell. Physiol..

[2] Mizgier M.L., Fernández-Verdejo R., Cherfan J., Pinget M., Bouzakri K., Galgani J.E. (2019). Insights on the Role of Putative Muscle-Derived Factors on Pancreatic Beta Cell Function. Front. Physiol..

[3] Boström P., Wu J., Jedrychowski M.P., Korde A., Ye L., Lo J.C., Rasbach K.A., Boström E.A., Choi J.H., Long J.Z. (2012). A PGC1-α-Dependent Myokine That Drives Brown-Fat-like Development of White Fat and Thermogenesis. Nature.

[4] Huh J.Y., Panagiotou G., Mougios V., Brinkoetter M., Vamvini M.T., Schneider B.E., Mantzoros C.S. (2012). FNDC5 and Irisin in Humans: I. Predictors of Circulating Concentrations in Serum and Plasma and II. mRNA Expression and Circulating Concentrations in Response to Weight Loss and Exercise. Metabolism.

[5] Moreno-Navarrete J.M., Ortega F., Serrano M., Guerra E., Pardo G., Tinahones F., Ricart W., Fernández-Real J.M. (2013). Irisin Is Expressed and Produced by Human Muscle and Adipose Tissue in Association with Obesity and Insulin Resistance. J. Clin. Endocrinol. Metab..

[6] Kim H., Wrann C.D., Jedrychowski M., Vidoni S., Kitase Y., Nagano K., Zhou C., Chou J., Parkman V.-J.A., Novick S.J. (2018). Irisin Mediates Effects on Bone and Fat via αV Integrin Receptors. Cell.

[7] Natalicchio A., Marrano N., Biondi G., Spagnuolo R., Labarbuta R., Porreca I., Cignarelli A., Bugliani M., Marchetti P., Perrini S. (2017). The Myokine Irisin Is Released in Response to Saturated Fatty Acids and Promotes Pancreatic β-Cell Survival and Insulin Secretion. Diabetes.

[8] Liu S., Du F., Li X., Wang M., Duan R., Zhang J., Wu Y., Zhang Q. (2017). Effects and Underlying Mechanisms of Irisin on the Proliferation and Apoptosis of Pancreatic β Cells. PLoS ONE.

[9] Zhang D., Xie T., Leung P.S. (2018). Irisin Ameliorates Glucolipotoxicity-Associated β-Cell Dysfunction and Apoptosis via AMPK Signaling and Anti-Inflammatory Actions. Cell. Physiol. Biochem..

[10] Liu C., Zhou J., Xu Y., Gong S., Zhu Y., Zhang H., Dong Y., Zhao B., Li X. (2022). Irisin Ameliorates Oxidative Stress-Induced Injury in Pancreatic Beta-Cells by Inhibiting Txnip and Inducing Stat3-Trx2 Pathway Activation. Oxid. Med. Cell. Longev..

[11] Ren Y., Zhang J., Wang M., Bi J., Wang T., Qiu M., Lv Y., Wu Z., Wu R. (2020). Identification of Irisin as a Therapeutic Agent That Inhibits Oxidative Stress and Fibrosis in a Murine Model of Chronic Pancreatitis. Biomed. Pharmacother..

[12] Han F., Ding Z.-F., Shi X.-L., Zhu Q.-T., Shen Q.-H., Xu X.-M., Zhang J.-X., Gong W.-J., Xiao W.-M., Wang D. (2023). Irisin Inhibits Neutrophil Extracellular Traps Formation and Protects against Acute Pancreatitis in Mice. Redox Biol..

[13] Norman D., Drott C.J., Carlsson P.-O., Espes D. (2022). Irisin—A Pancreatic Islet Hormone. Biomedicines.

[14] Rorsman P., Huising M.O. (2018). The Somatostatin-Secreting Pancreatic δ-Cell in Health and Disease. Nat. Rev. Endocrinol..

[15] Festing M.F.W. (2010). The Design of Animal Experiments. The UFAW Handbook on the Care and Management of Laboratory and Other Research Animals.

[16] Colaianni G., Cuscito C., Mongelli T., Pignataro P., Buccoliero C., Liu P., Lu P., Sartini L., Di Comite M., Mori G. (2015). The Myokine Irisin Increases Cortical Bone Mass. Proc. Natl. Acad. Sci. USA.

[17] Colaianni G., Mongelli T., Cuscito C., Pignataro P., Lippo L., Spiro G., Notarnicola A., Severi I., Passeri G., Mori G. (2017). Irisin Prevents and Restores Bone Loss and Muscle Atrophy in Hind-Limb Suspended Mice. Sci. Rep..

[18] Colaianni G., Errede M., Sanesi L., Notarnicola A., Celi M., Zerlotin R., Storlino G., Pignataro P., Oranger A., Pesce V. (2021). Irisin Correlates Positively With BMD in a Cohort of Older Adult Patients and Downregulates the Senescent Marker P21 in Osteoblasts. J. Bone Miner. Res..

[19] Storlino G., Colaianni G., Sanesi L., Lippo L., Brunetti G., Errede M., Colucci S., Passeri G., Grano M. (2020). Irisin Prevents Disuse-Induced Osteocyte Apoptosis. J. Bone Miner. Res..

[20] Alsharif K.F., Hamad A.A., Alblihd M.A., Ali F.A.Z., Mohammed S.A., Theyab A., Al-Amer O.M., Almuqati M.S., Almalki A.A., Albarakati A.J.A. (2023). Melatonin Downregulates the Increased Hepatic Alpha-Fetoprotein Expression and Restores Pancreatic Beta Cells in a Streptozotocin-Induced Diabetic Rat Model: A Clinical, Biochemical, Immunohistochemical, and Descriptive Histopathological Study. Front. Vet. Sci..

[21] Szymańczyk S., Kras K., Osiak-Wicha C., Kapica M., Puzio I., Antushevich H., Kuwahara A., Kato I., Arciszewski M.B. (2024). Immunodetection of Selected Pancreatic Hormones under Intragastric Administration of Apelin-13, a Novel Endogenous Ligand for an Angiotensin-like Orphan G-Protein Coupled Receptor, in Unweaned Rats. J. Vet. Res..

[22] Abo-Zeid F.S., Emam W.W.M. (2026). High-Dose Aluminium Chloride Exposure Disrupts the Renal Cortical Injury–Repair Balance in Rats: Partial Modulation by L-Carnitine Pretreatment. Animals.

[23] Cizkova K., Foltynkova T., Gachechiladze M., Tauber Z. (2021). Comparative Analysis of Immunohistochemical Staining Intensity Determined by Light Microscopy, ImageJ and QuPath in Placental Hofbauer Cells. Acta Histochem. Cytochem..

[24] Usborne A.L., Smith A.T., Engle S.K., Watson D.E., Sullivan J.M., Walgren J.L. (2014). Biomarkers of Exocrine Pancreatic Injury in 2 Rat Acute Pancreatitis Models. Toxicol. Pathol..

[25] Danese E., Montagnana M., Nouvenne A., Lippi G. (2015). Advantages and Pitfalls of Fructosamine and Glycated Albumin in the Diagnosis and Treatment of Diabetes. J. Diabetes Sci. Technol..

[26] Brunham L.R., Kruit J.K., Pape T.D., Timmins J.M., Reuwer A.Q., Vasanji Z., Marsh B.J., Rodrigues B., Johnson J.D., Parks J.S. (2007). Beta-Cell ABCA1 Influences Insulin Secretion, Glucose Homeostasis and Response to Thiazolidinedione Treatment. Nat. Med..

[27] Reaven E.P., Curry D.L., Reaven G.M. (1987). Effect of Age and Sex on Rat Endocrine Pancreas. Diabetes.

[28] Gannon M., Kulkarni R.N., Tse H.M., Mauvais-Jarvis F. (2018). Sex Differences Underlying Pancreatic Islet Biology and Its Dysfunction. Mol. Metab..

[29] Yong H.J., Toledo M.P., Nowakowski R.S., Wang Y.J. (2022). Sex Differences in the Molecular Programs of Pancreatic Cells Contribute to the Differential Risks of Type 2 Diabetes. Endocrinology.

[30] Gromada J., Franklin I., Wollheim C.B. (2007). α-Cells of the Endocrine Pancreas: 35 Years of Research but the Enigma Remains. Endocr. Rev..

[31] Quesada I., Tudurí E., Ripoll C., Nadal Á. (2008). Physiology of the Pancreatic α-Cell and Glucagon Secretion: Role in Glucose Homeostasis and Diabetes. J. Endocrinol..

[32] Merino B., Alonso-Magdalena P., Lluesma M., Ñeco P., Gonzalez A., Marroquí L., García-Arévalo M., Nadal A., Quesada I. (2015). Pancreatic Alpha-Cells from Female Mice Undergo Morphofunctional Changes during Compensatory Adaptations of the Endocrine Pancreas to Diet-Induced Obesity. Sci. Rep..

[33] Arrojo e Drigo R., Jacob S., García-Prieto C.F., Zheng X., Fukuda M., Nhu H.T.T., Stelmashenko O., Peçanha F.L.M., Rodriguez-Diaz R., Bushong E. (2019). Structural Basis for Delta Cell Paracrine Regulation in Pancreatic Islets. Nat. Commun..

[34] Svendsen B., Holst J.J. (2021). Paracrine Regulation of Somatostatin Secretion by Insulin and Glucagon in Mouse Pancreatic Islets. Diabetologia.

[35] Stolovich-Rain M., Enk J., Vikesa J., Nielsen F.C., Saada A., Glaser B., Dor Y. (2015). Weaning Triggers a Maturation Step of Pancreatic β Cells. Dev. Cell.

[36] Jacovetti C., Matkovich S.J., Rodriguez-Trejo A., Guay C., Regazzi R. (2015). Postnatal β-Cell Maturation Is Associated with Islet-Specific microRNA Changes Induced by Nutrient Shifts at Weaning. Nat. Commun..

[37] Puri S., Roy N., Russ H.A., Leonhardt L., French E.K., Roy R., Bengtsson H., Scott D.K., Stewart A.F., Hebrok M. (2018). Replication Confers β Cell Immaturity. Nat. Commun..

[38] Barsby T., Otonkoski T. (2022). Maturation of Beta Cells: Lessons from in Vivo and in Vitro Models. Diabetologia.

